# Exploration of nitrogen use efficiency and root-shoot-soil variation on the stoichiometric characteristics of carbon, nitrogen, phosphorous and potassium for winter wheat under various nitrogen treatments

**DOI:** 10.7717/peerj.20101

**Published:** 2025-10-03

**Authors:** Tianyu Lv, Shijie Liu, Suhao Lu, Chenyang Wang, Dongyun Ma, Guozhang Kang, Yingxin Xie, Jiaming Wu, Jutao Sun, Li-fang Wang

**Affiliations:** 1 College of Agronomy, Henan Agriculture University, Zhengzhou, China; 2 College of Tobacco Science, Henan Agriculture University, Zhengzhou, China

**Keywords:** Nitrogen application rate, Stoichiometric ratio, Yield, Nitrogen-use efficiency, Farmland ecosystem

## Abstract

**Background:**

Nitrogen (N) addition has significant effects on grain yield and nutrient use efficiency (NUE) in farmland ecosystems, and the total carbon: nitrogen: phosphorus: potassium (TC:TN:TP:TK) stoichiometry in plants and soil is of great significance for improving plant productivity and nutrition. However, there is still uncertainty regarding the effects of N fertilizers on the stoichiometric characteristics of different root-shoot parts of winter wheat during growth progression.

**Methods:**

‘Fengdecunmai5’ wheat was selected as the material for the positioning test field. Three N application treatments (0, 180 and 300 kg ha^−1^, designated N0, N180 and N300) were designed to study the yield, NUE, and stoichiometric characteristics of TC, TN, TP and TK in each organ at different stages, and to analyze the relationships among them.

**Results:**

The N180 treatment increased the total nitrogen (TN) content in roots, stems plus leaves and grain at each stage. The TN and TP content in stems plus leaves gradually decreased from jointing to maturity stages, whereas the TN content in grain gradually increased from filling to maturity stages. Under the N180 treatment, TN:TP values significantly increased, by 20.3%, 27.9% and 26.3% compared with N0 treatment, respectively. Moreover, the farmland ecosystem was mainly limited by the TN content. TK content in roots and TC:TN in stems plus leaves was significantly positively correlated with NUE. TN:TK and TN: TP in stems plus leaves were positively correlated with yield, and TN and TK content in stems plus leaves considerably influenced the yield.

**Conclusions:**

Under N180 treatment, compared with N0 and N300 treatments, wheat plants achieved higher nutrient uptake, and greater yield and NUE. This study contributes to the assessment of plant productivity and the precise optimization of ecosystem stoichiometry in the context of green sustainable agricultural development.

## Introduction

Wheat is an important food crop worldwide, and Henan Province is an important wheat-producing area in China, accounting for more than one-quarter of the country’s output, and therefore is particularly important to China’s food security (Wang et al., 2023). A large amount of nitrogen (N) fertilizer input is important to steadily increase the yield of winter wheat, but the N-recovery efficiency is only approximately 30% (Abubakar et al., 2023). Additionally, the unreasonable application of N fertilizer causes waste and many environmental problems, such as groundwater nitrate pollution and water eutrophication (Huang, Ju & Yang, 2017), which are contrary to the green sustainable development of agriculture. Consequently, reasonable N fertilizer management measures are particularly important for wheat stoichiometric ratios, high quality and high yields, as well as green production (Burton et al., 2024).

Ecological stoichiometry is the study of energy balance and the balance of multiple chemical elements in ecosystems, which can determine nutrient supply during plant growth (Zhu et al., 2020). The essence of the plant growth process is the accumulation of the most basic structural functional substances, such as total carbon (TC), total nitrogen (TN), total phosphorus (TP) and total potassium (TK), and the regulation of their relative proportions (Peng et al., 2016). N is both one of the nutrients for the growth and development of plants and a key structural component in plant tissiues. The application of N fertilizer directly affects the stoichiometric ratios of TC, TN, TP and TK in various parts of plants and the soil, thereby affecting crop growth and development, soil microbial activity and soil nutrient cycling (Elser et al., 2000), which in turn affect wheat grain yields. The nutrients TC, TN, TP and TK are essential for plants and the most basic components of ecosystems (Xing et al., 2021). The TC, TN and TP content in plants and their ratios can be used to assess the nutrient supply during plant growth (Zhu et al., 2020). Both TC:TN and TC:TP affect growth rates (Ågren, 2004) and can reflect the nitrogen-use efficiency (NUE) of crops. The TN:TP ratio is an indicator that limits productivity (Palpurina et al., 2018), and TK is an important element related to crop metabolism (Cui et al., 2019). A study on wheat fertilization strategies (Wang, 2016) has indicated that different levels of TN and TP in the soil significantly change the stoichiometric ratios in different wheat leaves under set soil TN and TP gradients; that the TP content of wheat leaves is relatively stable as the soil TN and TP content increases during each growth period; and that the application of TN and TP fertilizers significantly increases wheat yields.

To date, research on measurement ratios has mainly focused on the study of aquatic algae and terrestrial plants in natural ecosystems without artificial fertilization (Wang et al., 2018). However, few studies have examined the combined yield and NUE of cropland systems, and the stoichiometric ratios of TC, TN, TP and TK may vary depending on soil characteristics, crop types and ecological regions. The root system, an important organ enabling plants to absorb and transport mineral nutrients and water, drives plant growth and development (Ma, 1999). Wheat stems perform functions such as transport, support, storage and reproduction. The vascular bundle structure within stems is an important tissue transporting water and organic matter within organs (Feng, 2013). The growth conditions of roots and stems directly affect the growth and development of the aboveground parts and the final yield. Current studies on the changes in TC, TN, TP and TK content in crops have focused primarily on leaves and grains (Townsenet et al., 2007), as well as the effects of N on these organs. In contrast, studies on the roots and stems of wheat, which have functions including nutrient absorption, transport and storage, are relatively scarce (Zhao et al., 2021).

The trends in nutrient and TC:TN:TP:TK stoichiometric ratios, and their relationships with wheat yield and NUE of different organs at various growth stages and during the whole growth period, are not clear. This study was aimed at exploring the effects of N fertilizer on the content of TC, TN, TP and TK in wheat plants, as well as their stoichiometric ratios, and to analyze the role of N fertilization in the yield of the population and NUE. An additional aim was to analyze the relationships of the stoichiometric ratios of TC:TN:TP:TK with yield as well as NUE. We hypothesized that addition of an appropriate amount of N fertilizer would increase the total N content in plants and optimize the stoichiometric ratios of TC:TN:TP:TK in the farmland ecosystem for winter wheat, thereby enhancing the yield. The results will provide a basis for achieving high yields, efficient and appropriate N application rates, and the optimized wheat population structure.

## Materials **andMethods**

### Experimental site parameters

**Figure 1:**
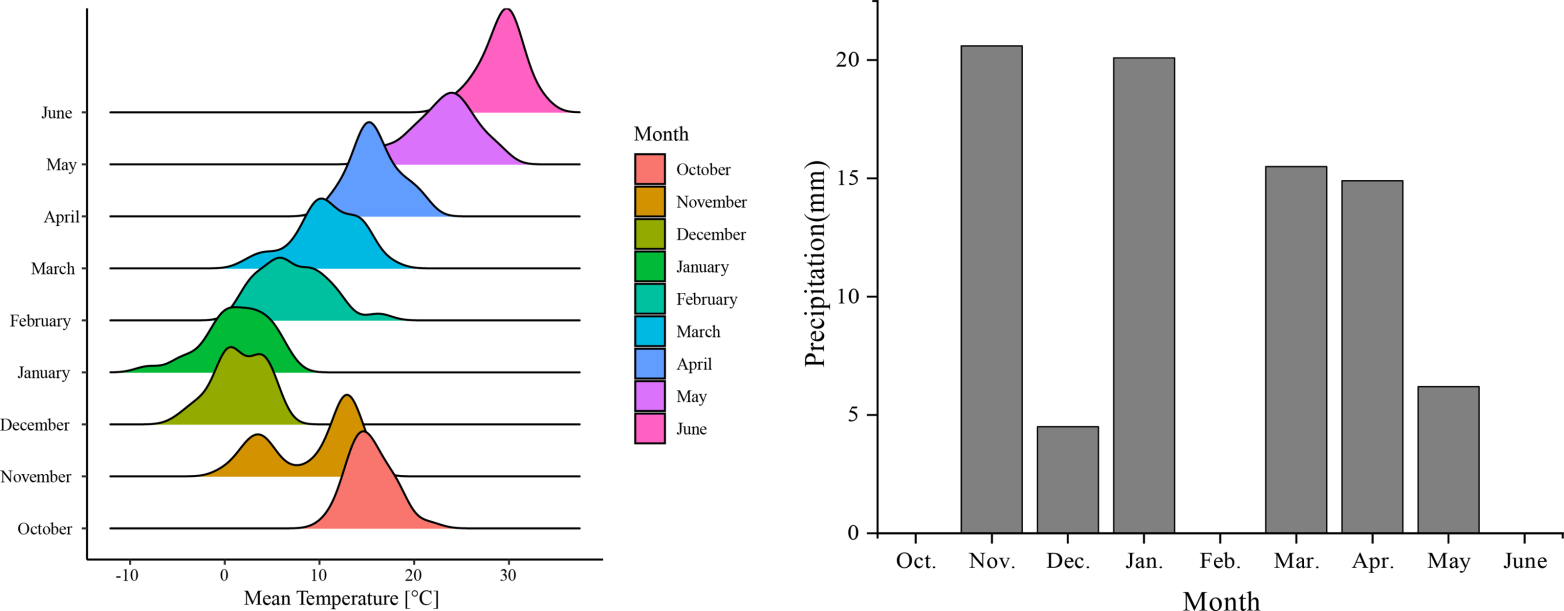
Monthly mean temperature and rainfall data from 2021–2022.

The 2 year field experiments were conducted at the Yuanyang Science and Education Demonstration Park of Henan Agricultural University (113°54′E, 35°5′N; 63.40 m above sea level), from 2020 to 2022. This area is a typical winter wheat area in the Huanghuai Region, which has a temperate continental monsoon climate. The annual average temperature is 15.7 °C, and the annual average precipitation is 556 mm. The total rainfall during the wheat growing seasons of 2020–2021 and 2021–2022 was 150.6 mm and 81.8 mm, respectively. The average monthly temperature and rainfall from 2021 to 2022 are shown in Fig. 1. The soil at the experimental site was cambisol, according to the World Reference Base for Soil Resources (Food and Agriculture Organization of the United Nations (FAO), 2014), and the 0–20 cm soil layer contained 16.3 g kg^−1^ organic matter, 0.87 g kg^−1^ TN, 0.57 g kg^−1^ TP, 0.41 g kg^−1^ TK, 111.1 mg kg^−1^ available N, 20.34 mg kg^−1^ available P and 137.27 mg kg^−1^ available potassium. The soil pH was 8.51, and the previous crop was maize.

### Experimental design

‘Fengdecunmai 5’ wheat was used in the experiment. The three tested N application levels (pure N) were 0 (N0), 180 (N180) and 300 kg ha^−1^ (N300), with three replicates per treatment. Half the N fertilizer was evenly applied before sowing the wheat, and the other half was top-applied during the jointing stage, with 150 kg ha^−1^ P_2_O_5_ and 120 kg ha^−1^ K_2_O as a single basal dressing before sowing. The applied N, phosphorus and potassium fertilizers were urea (46% N), concentrated superphosphate (46% P_2_O_5_) and potassium chloride (containing 60% of K_2_O). The row spacing was 20 cm, the basic seedlings were 3.23 million ha^−1^. Ridges separated the plots, and there were protective rows. The winter wheat was sown on October 23, 2020, and October 22, 2021, and harvested on June 2, 2021, and June 2, 2022, respectively. The manual pulling of weeds, and other agricultural measures were carried out in accordance with local conventional field management practices.

### Sample collection and measurements

For yield determination, when winter wheat was harvested, the spike number and grain number per spike were investigated in one m double rows, and then, three squares, each with an area of 4 m^2^ (2 m × 2 m), were randomly selected in each plot. The wheat ears were manually cut, threshed, and weighed, and the water content was measured and converted to the weight under the water content (12.5%). The 1,000-grain weights were investigated after harvest.

In wheat seedling stage, 1-m double rows with uniform growth were selected from each plot to investigate the population dynamics at the seedling, jointing, anthesis, filling and maturity stages. In total, 15 representative single stems were sampled at each growth stage (jointing, anthesis, filling and maturity). Then, the samples were classified into leaf, stem and spike (or glume and grain), placed in an oven at 105 °C for 30 min, and dried to a constant weight at 80 °C. The dry weight was used to calculate dry matter accumulation at each growth stage. The dry weight samples were ground into fine powders for analysis of N content. The N accumulation was calculated according to the dry matter weight of each plant part. The following formula was used to calculate NUE (Liu et al., 2023): NUE (kg kg^−1^) = grain yield/above-ground N accumulation.

At the jointing, anthesis, filling and maturity stages, soil–root samples were taken from the 0–30 cm soil layer with roots. The sample drill had an inner diameter of eight cm. First, scissors were used to cut off the aboveground portion of the plant, and then the drill was used to take a soil–root sample. Three drill samples were taken for each treatment: one in the wheat row, one in the middle of two rows of wheat and one tangential to the wheat row. The three samples were combined into one soil–root sample, with three repetitions per treatment. The samples were placed into a 60 mesh bag and rinsed repeatedly with low-pressure tap water until only the roots and impurities remained in the bag. The remains were poured into a basin, and the impurities, such as straw floating on the water, were removed. The roots and soil that had sunk in the bottom of the basin were separated with pointed tweezers, and the selected roots were stored in the refrigerator in a self-sealing bag for the determination of roots morphological indicators.

The five-point sampling method was used to collect soil samples. This method can cover field variations by dispersing sampling points, while avoiding deviations caused by local fertilization abnormalities (such as fertilizer residues or uneven root distribution) and decreasing the effects of soil spatial heterogeneity on the results. A sterilized shovel was used to dig 30 cm from 5 cm away from each wheat plant. According to the method described by Ren et al. (2024), loose soil was removed by gentle shaking of the root systems, and soil covering the surfaces of the wheat roots was removed with a disinfected brush. The mixed soil samples were then air-dried, and visible plants, root materials, and pebbles were removed. The air-dried soil samples were crushed and sieved to one mm, then used to test soil SOC, TN, TP and TK.

The TN content was determined using an Automatic Kjeldahl Apparatus K1100-K1100F (Hanon Technologies, Jinan, China). The TC content was determined using the potassium dichromate volumetric method (Yang et al., 2024). The TP and TK concentrations were obtained using the HF-HClO_4_-HNO_3_ digestion method with a 6300 ICP-AES (Thermo Scientific, Waltham, MA, USA) (Li, Wang & Wang, 2024).

The effects of nutrient content and nutrient metrological ratios on yield and NUE were assessed using random forest models. The variables selected included roots, stems plus leaves, nutrient content (TC, TN, TP and TK) and nutrient stoichiometric ratios (TC:TN, TC:TP, TC:TK, TN:TP, TN:TK and TP:TK). With the random forest algorithm—which extends classification and regression tree (CART) approaches and handles nonlinear, nonadditive relationships—we quantified the relative importance of environmental variables. The percentage increase in mean squared error (%IncMSE), according to the randomForest package in R, was used. The significance of the effect of each environmental variable was recorded using the ‘rf Permute’ package. The % Inc MSE was used as the criterion for evaluating the relative importance of factors affecting wheat grain yield and NUE.

### Statistical analyses

Microsoft Office Excel (version 2016 for Windows, Microsoft Office Software, Redmond, WA, USA) was used for data collation. SPSS (version 26.0 for Windows, SPSS Software, Chicago, IL, USA) was used for data analyses. The normality of the data was assessed with the Shapiro–Wilk test, and the homogeneity of variance was verified with Levene’s test. The one-way ANOVA test was used to examine the differences among various treatments, and Duncan’s method was used for multiple comparisons. Origin (version 2021 for Windows, Origin Software, Northampton, MA, USA) and R (v. 4.1.1; R Core Team, 2021) were used to create the figures.

## Results

### Yield and NUE

**Figure 2:**
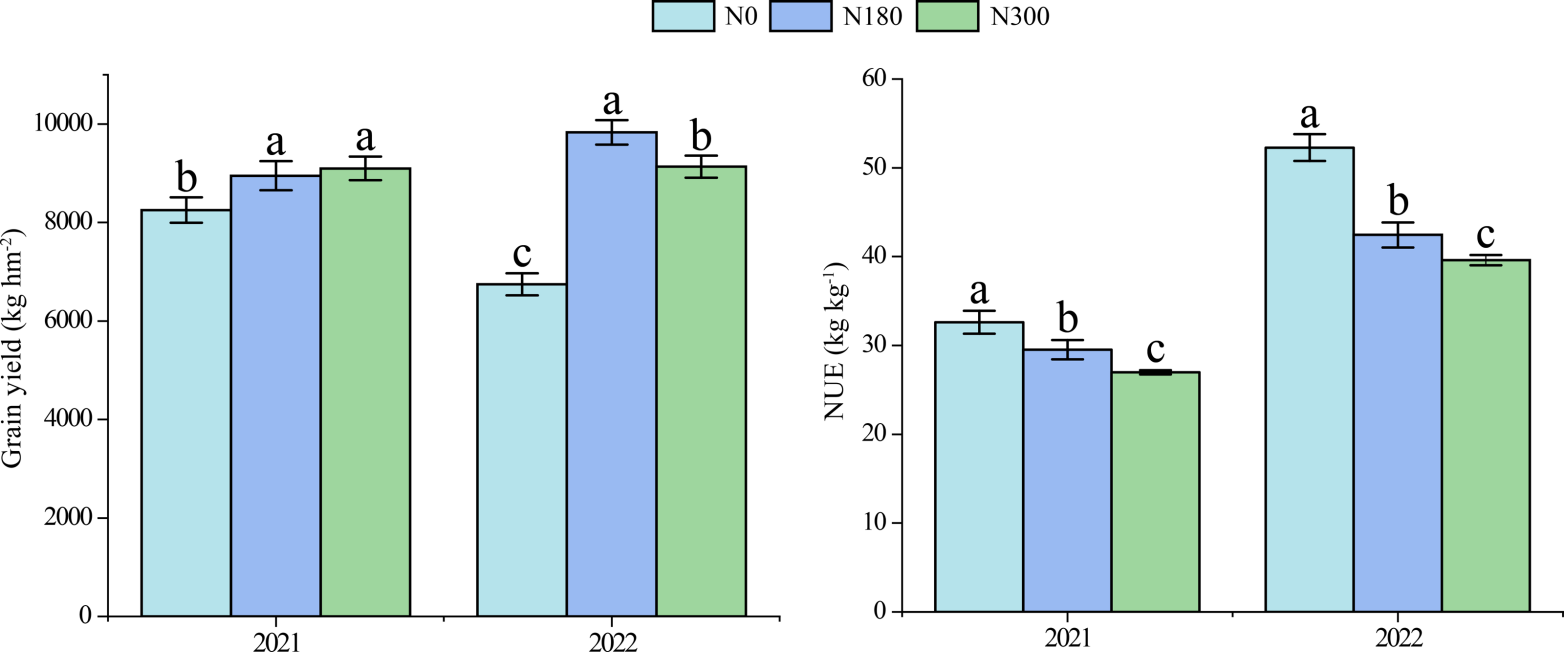
Winter wheat yields and NUE values under different N treatments in 2021 and 2022. NUE: nitrogen-use efficiency; N0, N180 and N300: N application rates of 0, 180 and 300 kg hm^−2^, respectively.

In 2021, the winter wheat yield increased along with N application rate, reaching 9,097.69 kg ha^−^^1^ under N300 treatment (Fig. 2), which was a significant increase of 10.3% with respect to N0 treatment. In 2022, with increasing N application rate, the yield first increased and then decreased, and the maximum yield reached 9,831.80 kg ha^−1^ under N180 treatment, thereby indicating a significant increase of 45.8% with respect to N0 treatment. In both 2021 and 2022, NUE decreased significantly as the N application increased.

### Effects of different N treatments on the **TC, TN, TPandTK contentin different parts of winter wheatat variousgrowth stages**

The TC, TN, TP and TK content in winter wheat roots, stems plus leaves and grain under different N fertilizer treatments during different stages is shown in Table 1. In wheat roots during the jointing stage, the TC content under N180 treatment was the highest, significantly increasing by 6.1% compared with N0 treatment, whereas at the anthesis stage, as the N application increased, the TC content showed a decreasing trend. It was lowest under N300 treatment. The TN content in wheat root systems increased significantly under N180 treatment. With an increasing N application rate, root TP content was highest under N300 treatment, representing a significant increase of 20.4% with respect to N0 treatment. During the jointing stage, the root TK content first decreased and then increased with increasing N application rate; the N180 treatment significantly decreased by 28.4% compared with N0 treatment. During the anthesis stage, root TK content under N180 treatment significantly decreased, by 40.0% with respect to N0 treatment, and during the filling stage, root TK content first significantly increased and then decreased with increasing N application rate. The N180 treatment was associated with the highest TK content and was significantly increased, by 44.7% with respect to N0 treatment.

**Table 1:** The C, N, P and K content in different winter wheat parts during various growth stages under different N treatments.

Index	Treatment	Root (g kg^−1^)				Stems and leaves (g kg^−1^)				Grain (g kg^−1^)	
		Jointing stage	Anthesis stage	Filling stage	Maturity stage	Jointing stage	Anthesis stage	Filling stage	Maturity stage	Filling stage	Maturity stage
TC	N0	407.98 b	461.83 a	429.55 a	435.86 a	436.91a	393.69 b	419.48 a	405.30 a	452.47 a	435.64 b
	N180	432.92 a	433.07 b	460.91 a	435.64 a	431.25 a	408.67 b	416.93 a	403.74 a	454.76 a	452.02 a
	N300	425.39 a	414.33 b	434.11 a	449.35 a	448.64 a	431.44 a	396.23 a	425.22 a	446.60 a	447.30 ab
TN	N0	5.38 b	3.65 b	5.92 b	4.67 c	24.73 b	13.01 b	10.57 b	4.51 b	17.64 a	20.63 b
	N180	6.56 a	4.75 a	7.31 a	6.96 a	30.10 a	16.16 a	14.12 a	4.78 b	18.05 a	28.45 a
	N300	5.54 ab	4.48 a	7.21 a	5.86 b	28.18 a	18.00 a	13.00 a	6.00 a	19.87 a	24.07 ab
TP	N0	1.48 a	1.36 b	1.42 a	1.36 a	3.24 a	2.24 a	1.98 a	1.17 a	4.11 a	4.03 a
	N180	1.61 a	1.43 b	1.43 a	1.40 a	3.01 a	2.54 a	1.83 a	0.97 b	3.96 a	3.85 a
	N300	1.54 a	1.64 a	1.39 a	1.34 a	3.22 a	2.47 a	1.94 a	1.22 a	4.08 a	3.83 a
TK	N0	4.74 a	4.09 a	2.98 b	2.63 a	37.46 a	21.35 b	25.38 a	25.87 a	49.12 a	9.33 a
	N180	3.39 c	2.46 b	4.31 a	2.62 a	42.53 a	23.53 ab	31.12 a	26.93 a	48.06 a	7.87 a
	N300	3.94 b	2.76 b	2.22 c	2.76 a	39.00 a	27.59 a	29.79 a	29.02 a	49.52 a	8.23 a

**Notes.**

TC Total carbon content TN Total nitrogen content TP Total phosphorus content TK Total potassium content

Different lowercase letters indicate significant differences between different treatments (*P* <0.05).

The TN content of stems plus leaves under N180 treatment was higher than observed with N0 treatment at all stages, and significantly increased, by 21.7% and 36.6% with respect to N0 treatment at the jointing and filling stages, respectively. Overall, the TN and TP content in stems plus leaves gradually decreased from jointing to maturity. The TP content under N180 treatment significantly decreased, by 17.4% with respect to N0 treatment during the maturity stage. The TK content in stems plus leaves increased gradually with N application.

At the maturity stage, under N180 treatment, compared with N0 treatment, the TC and TN content in grain significantly increased, by 3.8% and 37.9%, respectively. The TK content in grain during the maturity stage was significantly lower than that during the filling stage.

### Effects of different N treatments on C, N, P and K **contentinvarious plant organsduring the whole growth period**

**Figure 3:**
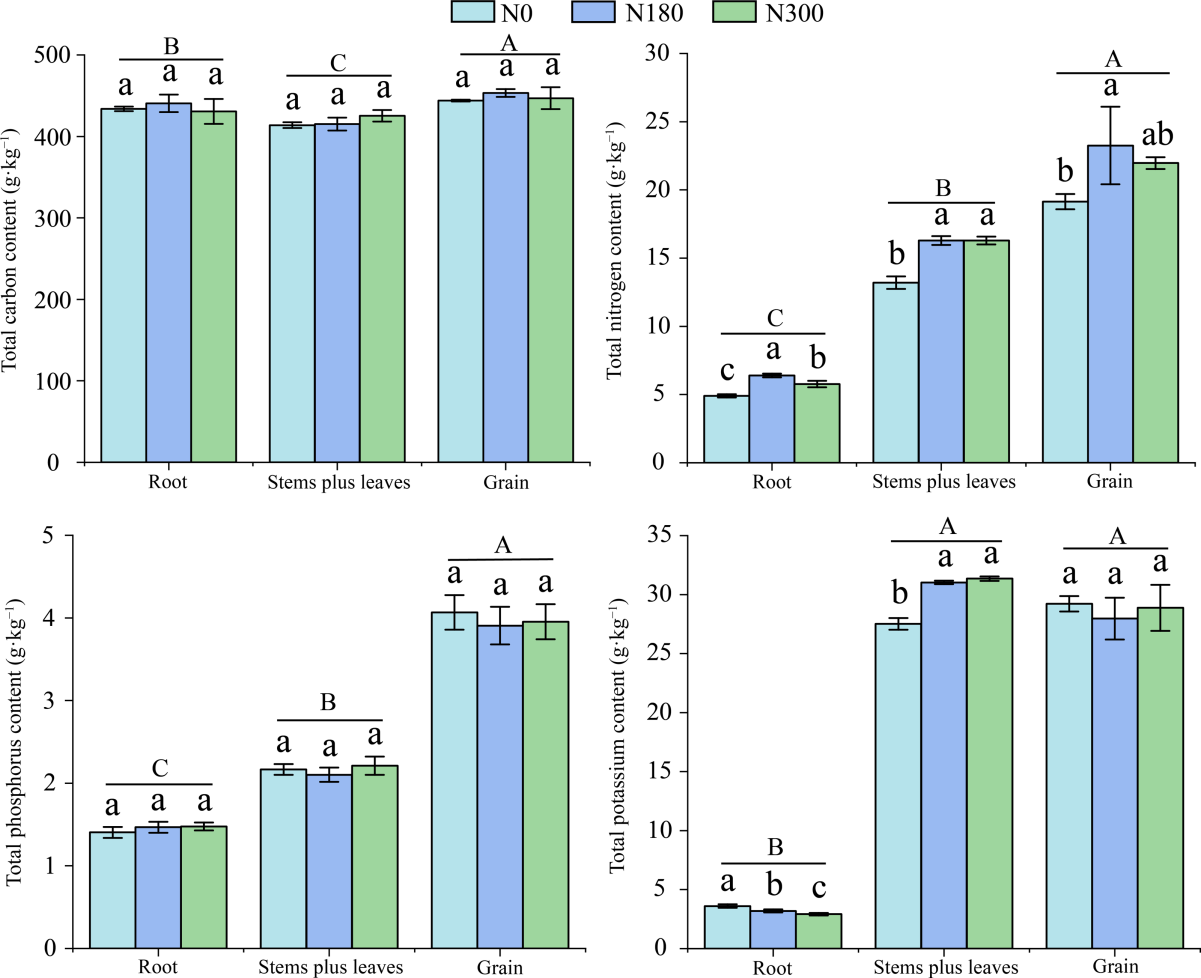
The carbon (C), nitrogen (N), phosphorous (P) and potassium (K) content in various parts of winter wheat during the entire growth period under different N treatments. Different uppercase and lowercase letters indicate significant differences among different plant parts and different treatments, respectively (*P* < 0.05).

As shown in Fig. 3, the TC and TP content in wheat roots, stems plus leaves and grain showed no significant changes with increasing N application rate. However, the TC and TP content in different parts varied significantly, and the TC content in grain and stems plus leaves was 435.10–461.52 g kg^−1^ and 406.29–433.11 g kg^−1^, respectively. The high TP content in grain was 3.73–4.21 g kg^−1^, and the low TP content in roots was 1.34–1.52 g kg^−1^.

The TN content in roots, stems plus leaves and grain significantly differed, and the highest and lowest TN content were observed in grain at 25.55 g kg^−1^ and in roots at 4.76 g kg^−1^. Under N180 treatment, the TN content in roots, stems plus leaves and grain was significantly greater (30.4%, 23.4% and 21.5%, respectively) than observed with N0 treatment.

With an increasing N application rate, the TK content in wheat roots significantly decreased. The TK content in stems plus leaves under N300 treatment significantly decreased, by 19.1% with respect to the N0 treatment, but it significantly increased, by 13.9%, under N180 treatment compared with N0 treatment. The TK content in stems plus leaves and grain was significantly higher than observed in roots, reaching as high as 21.00–31.57 g kg^−1^ in stems plus leaves compared with only 2.85–3.75 g kg^−1^ in roots.

### Effects of different N treatments on stoichiometric ratios of **plant organsduring the whole growth period**

**Figure 4:**
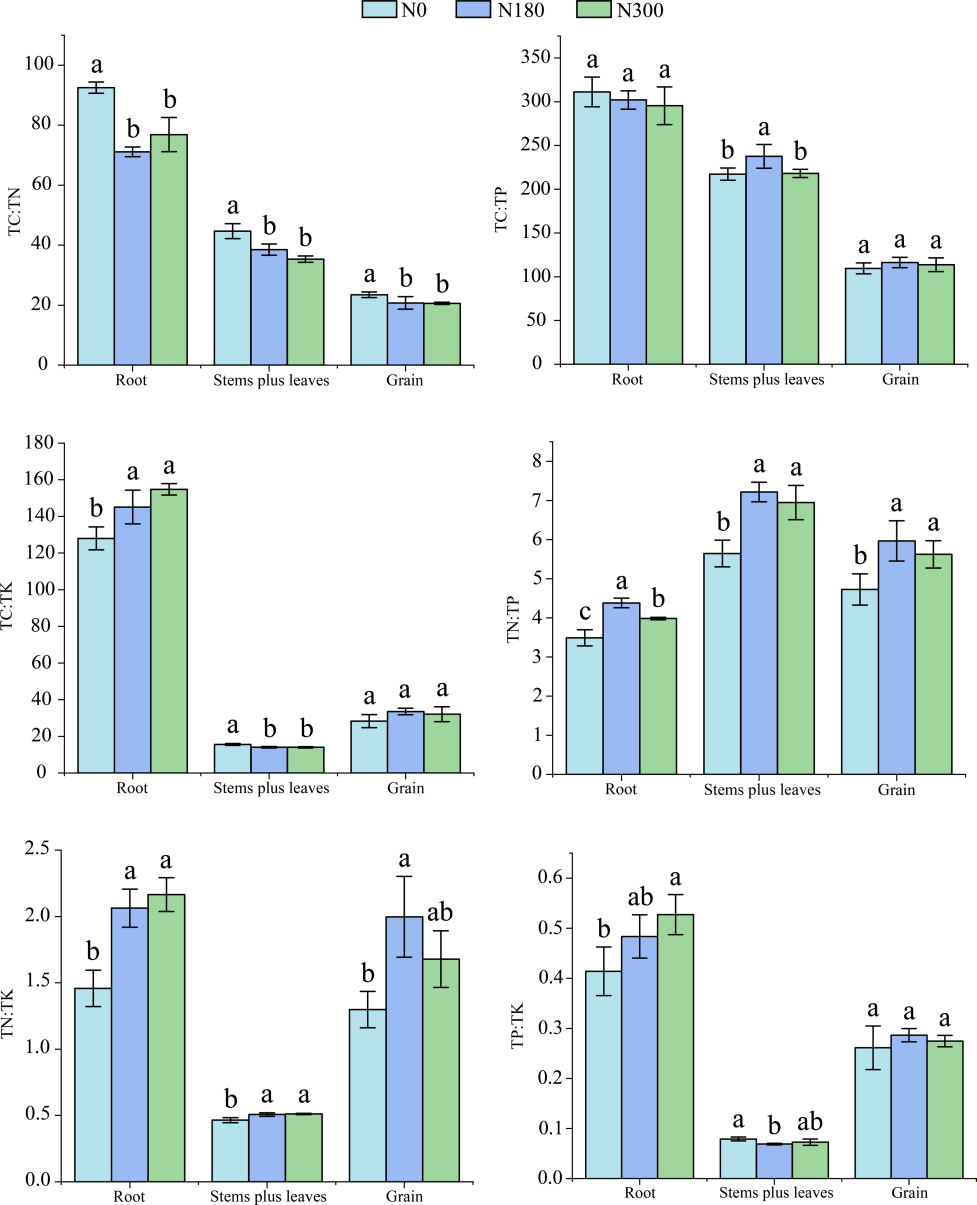
Chemical stoichiometry ratios of different plant organs during entire growth period under different N treatments. Different lowercase letters indicate significant differences in the same plant part among different treatments (*P* < 0.05).

As shown in Fig. 4, TC:TN and TC:TP values in wheat roots, stems plus leaves and grain gradually decreased. The TC:TK, TN:TK and TP:TK values were the highest in roots, followed by grain, and the lowest in stems plus leaves. The TN:TP value was the highest in stems plus leaves, followed by grain and roots.

With increasing N application rate, the TC:TN value in wheat roots under N180 treatment significantly decreased, by 23.1% with respect to N0 treatment, whereas TC:TK, TN:TK and TP:TK values gradually increased. Compared with N0 treatment, N300 treatment significantly increased TC:TK, TN:TK and TP:TK values, by 20.9%, 48.6% and 27.3%, respectively, whereas the N180 treatment significantly increased the TN:TP value, by 20.3%.

Under N300 treatment, the TC:TN and TC:TK values in wheat stems plus leaves showed gradual decreasing trends that resulted in significant decreases of 20.9% and 10.4%, respectively, with respect to N0 treatment, as the N application rate increased. Compared with N0 treatment, N180 treatment significantly increased TC:TP and TN:TP values in stems plus leaves, by 9.3% and 27.9%, respectively, and the TN:TK value under N300 treatment was as much as 10.1% greater than observed with N0 treatment. However, compared with N0, N180 treatment significantly decreased the TP:TK value in stems plus leaves, by 12.7%.

The TC:TN value in wheat grain decreased gradually as the N application rate increased; a minimum decrease of 12.3% was observed under N300 treatment compared with N0 treatment. The highest TN:TP and TN:TK values in grain under N180 treatment showed significant increases, of 26.3% and 52.3%, respectively, with respect to N0 treatment.

### Relationship between NUE and stoichiometric ratios of **various plant organs**

**Figure 5:**
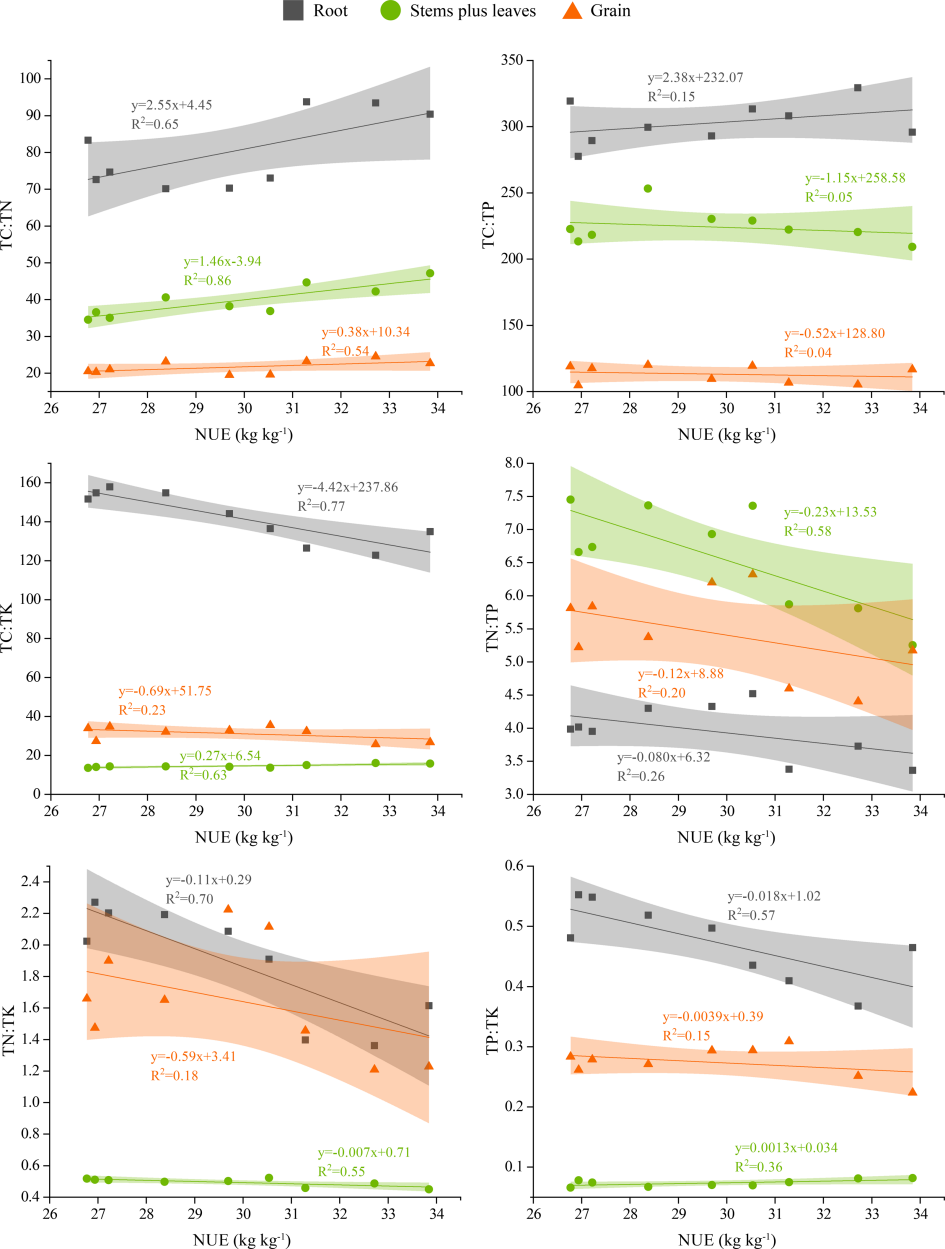
Correlations between winter wheat NUE and stoichiometric ratios in different plant parts. The shaded area represents the scatter plot 95% confidence interval.

As shown in Fig. 5, the relationships between stoichiometric ratios in winter wheat roots, stems plus leaves, grain and wheat NUE, were expressed by correlation equations and R^2^ values. They revealed that TC:TN values in wheat roots, stems plus leaves and grain were positively correlated with NUE, whereas the TC:TP value was positively correlated with roots and negatively correlated with stems plus leaves and grain. The TC:TK and TP:TK values in roots and grain were negatively correlated with NUE, whereas the TC:TK and TP:TK values in stems plus leaves were positively correlated with NUE. Additionally, the TN:TP and TN:TK values in roots, stems plus leaves and grain were negatively correlated with NUE.

### Relationship between **grainyield and stoichiometric ratios ofvariousplantorgans**

**Figure 6:**
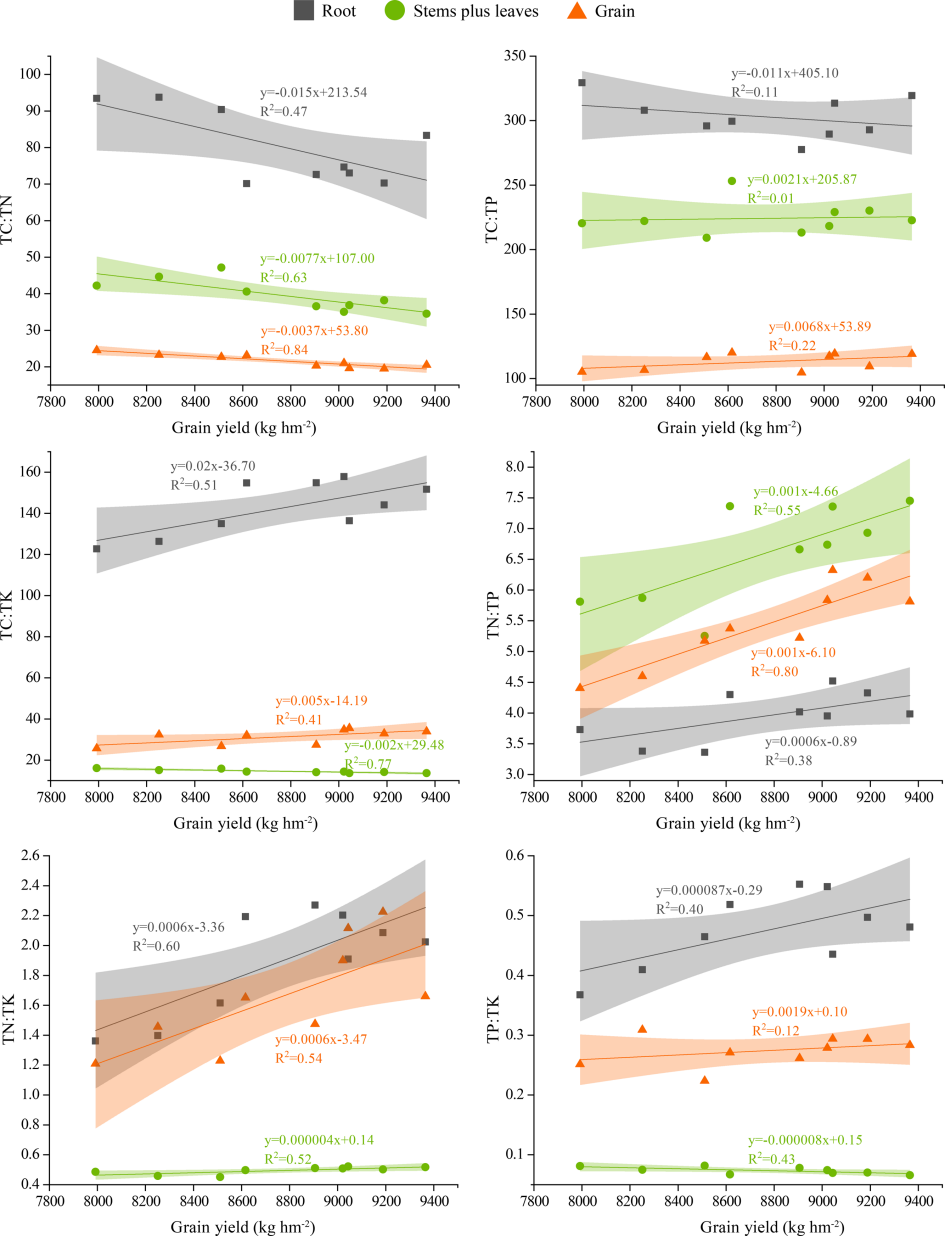
Correlations between gain yield and the stoichiometric ratios of different plant organs. The shaded area represents the scatter plot 95% confidence interval.

The correlation equations and R^2^ values between the stoichiometric ratios of winter wheat roots, stems plus leaves and grain and wheat yield are shown in Fig. 6. The TC:TN values in roots, stems plus leaves and grain were negatively correlated with yield, the TC:TP value in roots was negatively correlated with yield, and the TC:TP values in stems plus leaves and grain were positively correlated with yield. The TC:TK and TP:TK values in roots and grain were positively correlated with yield, whereas the values in stems plus leaves were negatively correlated with yield. The TN:TP and TN:TK values in roots, stems plus leaves and grain were positively correlated with yield, and the TN:TP value in stems plus leaves and grain were almost consistent with the yield slope. Additionally, the TN:TK values in roots and grain were consistent with the yield slope.

### Correlations among soil indexes, yield, NUE and stoichiometric ratios of **variousplant organs**

**Figure 7:**
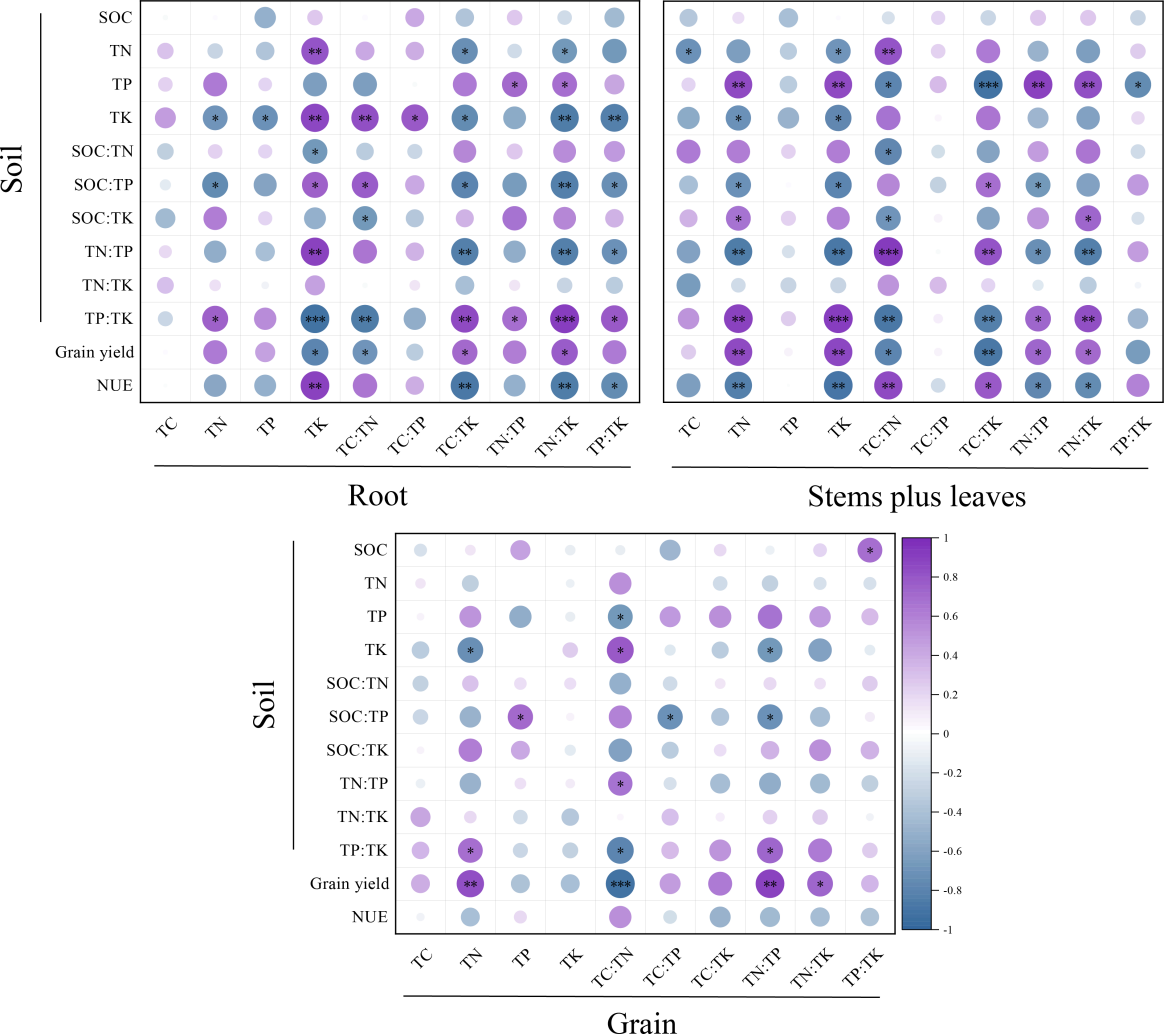
Correlation analysis of SOC, N, P and K content, stoichiometric ratio, yield and NUE with C, N, P and K content and stoichiometric ratios in different winter wheat parts. Asterisks (*, **, ***) indicate significant differences at probability levels of 0.05, 0.01 and 0.001, respectively. The purple circles represent positive correlations, whereas the blue circles represent negative correlations.

As shown in Fig. 7, soil organic carbon (SOC) was significantly positively correlated with the TP:TK value in grain (*P* < 0.05). The TN content was significantly positively correlated with the TK content in roots and the TC:TN value in stems plus leaves (*P* < 0.01). It was significantly negatively correlated with the TC:TK value and the TN content in roots, as well as the TC and TK content in stems plus leaves (*P* < 0.05). The correlation between the TP content in soil and stems plus leaves was greater, and the correlation between the TK content in soil and roots was greater. However, the correlations between the TK content in soil and the TN content in roots, stems plus leaves and grain were significantly negative (*P* < 0.05). The SOC:TN value in soil was significantly negatively correlated with the TK content in roots and the TC:TN content in stems plus leaves (*P* < 0.05). The SOC:TP value was significantly negatively correlated with the TN content and TC:TK, TN:TK and TP:TK values in roots, the TN and TK content and the TN:TP value in stems plus leaves and the TC:TP and TN:TP values in grain. There was a significant positive correlation between the TC:TK value in stems plus leaves and the TP content in grain (*P* < 0.05).

Yield was significantly positively correlated with the TN content in stems plus leaves and grain (*P* < 0.01), significantly negatively correlated with the TK content in roots (*P* < 0.05) and significantly positively correlated with the TK content in stems plus leaves (*P* < 0.01). The yield was significantly negatively correlated with the TC:TN values in roots, stems plus leaves and grain, significantly positively correlated with the TN:TK value and significantly negatively correlated with the TC:TK value in stems plus leaves (*P* < 0.01).

The NUE was significantly positively correlated with the TK content in roots and the TC:TN value in stems plus leaves (*P* < 0.01), significantly positively correlated with the TC:TK value in stems plus leaves (*P* < 0.05) and significantly negatively correlated with the TC:TK, TN:TK and TP:TK values in roots as well as the TN and TK content and the TN:TP and TN:TK values in stems plus leaves. On the whole, soil indexes, yield and NUE were more correlated with stoichiometric ratios in winter wheat roots and stems plus leaves than grain.

### Analysis of the importance of nutrient **contentand nutrient stoichiometric ratios to yield and NUE in different winter wheat parts during the whole growth period**

**Figure 8:**
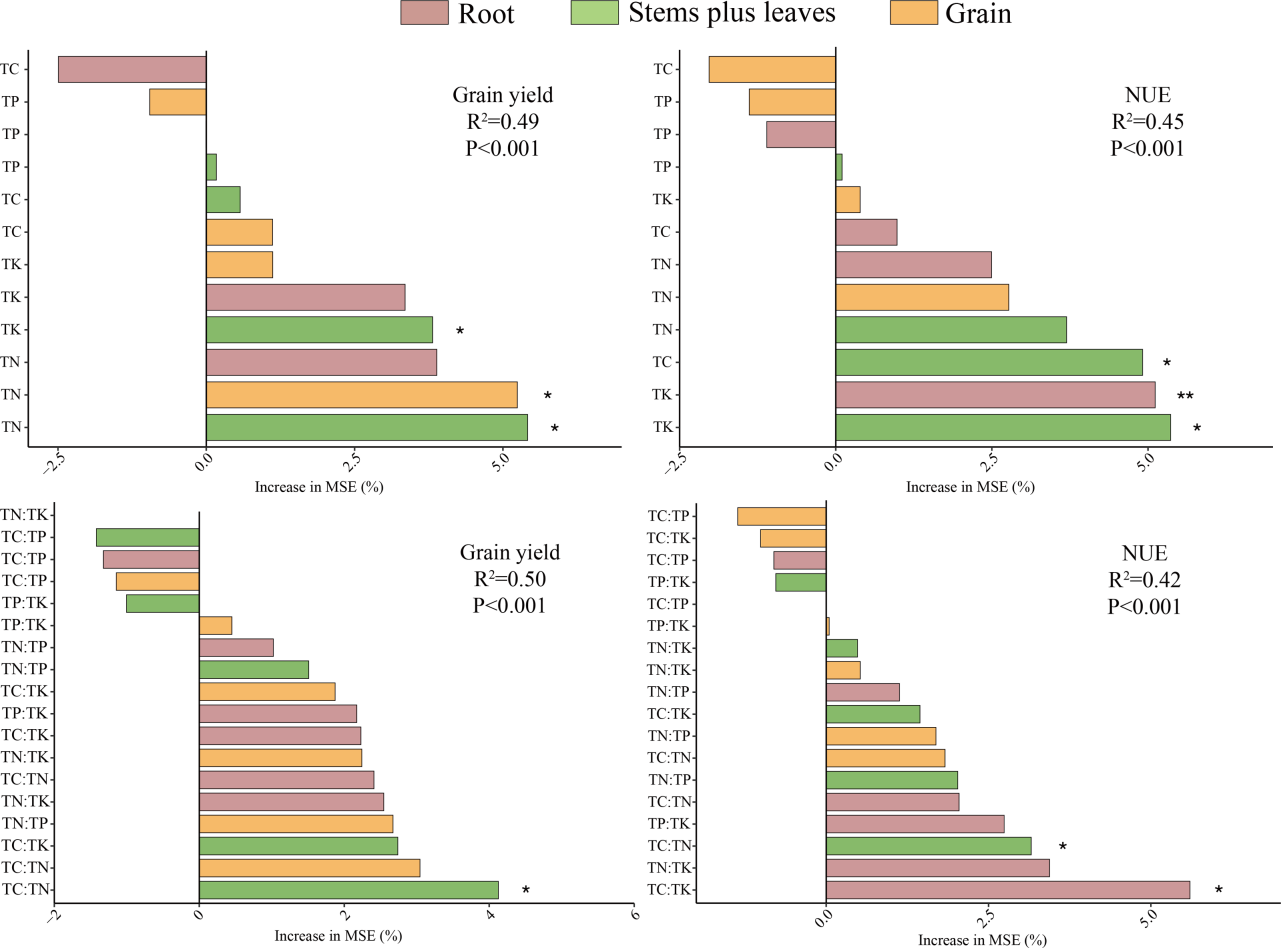
Nutrient content and stoichiometric comparisons of nutrients in different winter wheat parts during the entire growth period: importance to yield and NUE. Asterisks (*, **) indicate significant differences at probability levels of 0.05 and 0.01, respectively.

The importance levels of the TN content in stems plus leaves, grain and roots, and the TK content in stems plus leaves and roots, in winter wheat yield (5.4%, 5.2%, 3.9%, 3.8% and 3.4%, respectively; Fig. 8) was greater than those of the TP and TC content. The TK content in stems plus leaves and roots, and the TC and TN content in stems plus leaves, had greater influence on the NUE (5.4%, 5.1%, 4.9% and 3.7%, respectively). The TC:TN value in stems plus leaves was most important for wheat yield, at 4.13%, whereas the importance levels of the TC:TK value in roots; the TN:TK value in stems plus leaves; and the TC:TN value in stems plus leaves were greater for NUE (5.6%, 3.4% and 3.2%, respectively). In general, the nutrient content and stoichiometric ratios in stems plus leaves and grain had greater impacts on the yield of winter wheat, whereas the nutrient content and stoichiometric ratios in stems plus leaves and roots had greater impacts on NUE.

## Discussion

### Effects of N application rate on root-shoot **nutrient content, yield and NUE**

N accumulation and translocation in wheat organs can reflect N absorption and plant growth (Zheng et al., 2020), thus affecting NUE and ultimately enhancing yield (Li et al., 2023). The addition of N fertilizers can compensate for deficiencies in soil nutrients, thereby increasing the absorption and assimilation of soil TC, TN, TP and TK by plants, and consequently affecting crop growth and development (Lu et al., 2019). TC, TN, PT, TK and other elements are important for photosynthesis and the synthesis of genetic materials in plants; consequently, they provide nutritional protection for plant growth and development (Kaur et al., 2023). Leaves are the main organs for material and energy exchanges between plants and the environment, with the distributions of nutrients in the soil being higher (Zhang et al., 2023). In this study, TN, TP and TK content in the upper plant parts were significantly higher than those in the root system. The N and P content of plants varies greatly across growth stages (Han et al., 2005). In this experiment, the TN content in stems plus leaves decreased significantly from 27.67 g kg^−1^ at the jointing stage to 5.09 g kg^−1^ at the maturity stage. In our study, N addition significantly increased the TN content in wheat organs, but the TN content in grain was higher than observed in stems plus leaves and roots. The TN content in wheat grain increased significantly with increasing amounts of N fertilizer, and wheat growth promoted the redistribution of TN to grain organs, thus resulting in a dilution effect, which produced relatively low TN content in stems plus leaves and roots. These results were similar to those of Nan et al. (2017). The content of various elements in crop organs is related to their own physiological structures and synthesis, and also shows characteristic responses to the soil nutrient environment (Viciedo et al., 2021). N addition had no significant effects on the TC and TP content in wheat plants, thus indicating that N addition might increase the supply pressure on soil TC and TP. The increase in ammonium ions in soil caused by N addition may inhibit the absorption of TC and TP by plants (Wang, Jensen & Magid, 2016). N addition reduced the TK content in wheat roots and increased the TK content in stems plus leaves. Studies have found that if the level of a certain element in a plant is proportional to the supply capacity of the element in the soil, then the growth of the plant is limited by this element (Garnier, 1998). In this study, there was a significant positive correlation between the TK content in root and the TK content in soil. This indicated that wheat growth was limited by the soil TK level. Additionally, N application significantly increased wheat yield, but NUE significantly decreased. These results were consistent with those of Shi et al. (2021), indicating that an appropriate N fertilizer treatment was conducive to increasing wheat yield.

### Effects of N application rate on plant nutrient metrological **ratiocharacteristics**

Crops usually have the ability to adjust their own elemental stoichiometric characteristics (Yuan et al., 2019). During changes in environmental conditions, plants can actively adjust the content and relative abundance of nutrient elements in their tissues, the ecological stoichiometric characteristics of the nutrients (Li et al., 2023). As the basic components of organic matter, TC and TN form a large and complex TC and TN cycle, that controls the plant TC, TN and TP ratios in the ecosystem, which may reflect the growth rates and NUE of plants (Fang et al., 2023). As the most basic physiological metabolisms in wheat growth and development, TC and TN metabolism are important indicators reflecting wheat’s physiological status, growth activity and disease resistance (Zhang et al., 2021). The elemental stoichiometric ratios in plants clearly indicate any nutritional deficiencies, and TC:TN and TC:TP values represent their assimilation capabilities and can reflect the levels of their nutrient utilization, which is of great ecological significance (Deng et al., 2019). In this study, stems plus leaves TN:TK markedly influenced NUE.

The TN:TP, TN:TK and TK:TP values can be used as indicators of plant growth restriction (Li, Wang & Wang, 2024). For optimal plant growth, the literature has suggested the following thresholds: TN:TP ratios of 10–14 typically indicate balanced N and P supply (Güsewell, 2004), whereas TN:TK < 2.1 and TK:TP > 3.4 reflect sufficient K availability (Wu et al., 2019). TN:TP may be used to represent the environmental nutrient supply for plant growth (Güsewell & Bollens, 2003). Low TN:TP generally indicates that the community lacks TN, and a high TN:TP indicates that plant productivity is limited by TP. Braakhekke & Hooftman (1999) have suggested that when the TN:TP value is greater than 14 and the TP content in plant leaves is lower than 1.0 g kg^−1^, the system is limited by TP. When the TN:TP value is less than 10 and the TN content of plant leaves is less than 20.0 g kg^−1^, the ecosystem is limited by TN. A TN:TP value in the range of 10 to 14 is considered to represent limitation by both elements (TN <  20.0 g kg^−1^, TP <  1.0 g kg^−1^) or neither element (TN > 20.0 g kg^−1^, TP >  1.0 g kg^−1^). When TN:TK >  2.1 and TK:TP <  3.4, plant growth is limited primarily by TK (Wu et al., 2019). In this study, the TC:TN value decreased and the TN:TP and TN:TK values increased in wheat organs as the amount of the N application increased. Qin et al. (2022) showed in a study of water-N coupling on cumulative distributions and stoichiometric characteristics of TC, TN and TP in rice in black soil that N application treatments increased TN content but had relatively little effect on TC content in the plants. The TC:TN value of rice plants decreased and the TN:TP value of rice plants increased, in agreement with the results of this study. Nan et al. (2017) found in their study on spring wheat in the semi-arid hilly and gully region of the Loess Plateau in Longzhong that the combined application of controlled release biomass TC and TN fertilizer increased the TN content in wheat organs in the soil to varying degrees; increased the TC, TN and TP nutrient content in wheat; and decreased the TC:TN, TC:TP and TN:TP values in plants. These results are not completely consistent with the results of this study. Similar results have been reported elsewhere for various plants, probably due to different years (different environmental conditions) and the timing of crop development stages (Ågren, 2008; Elser et al., 1996; Malhi et al., 2006; Ye et al., 2014; Zhong et al., 2015). Findings from Yan, Zhong & Shangguan (2015) on the metrological ratios of wheat under fertilization measures in the Loess Plateau were consistent with the results of this study. With increasing N fertilizer dose, the wheat stalk and grain TN content increased significantly, whereas the TP content showed a downward trend, and the N:P value increased significantly.

### Relationships among **nutrient metrological ratios of root-shoot-soil nutrients, yield and NUE**

Terrestrial plants primarily draw nutrients from the soil, and the nutrient content in the above-ground parts of plants is usually related to the soil nutrient content. In contrast, the soil-forming characteristics are easily affected by factors such as soil parent material, climate and landform, resulting in large differences in soil nutrient elements under different environments, which affects the elemental stoichiometric ratios of plants (Lin et al., 2019). The nutrients in soil that can be utilized by plants have direct effects on the elemental compositions and content in plant leaves (Zhao et al., 2024), and consequently affect plants’ TN:TP and TC:TP values and growth rates (Chen, Wang & Baoyin, 2021). In this study, the TN content in the soil was significantly positively correlated with the TK content in roots and the TC:TN value in stems plus leaves. The TP content in the soil was significantly positively correlated with the TN and TK content in stems plus leaves and the TC:TP values in roots and stems plus leaves. These results indicated that soil nutrients are involved in plant growth and development, as important sources of functional operations (Pan et al., 2022). When *Arabidopsis thaliana* is limited by the TN content in the soil, its growth rate increases along with the leaf TN:TP value, whereas when it is limited by TP, the growth rate decreases as the leaf TN:TP value increases (Yan et al., 2015). Similarly, under the condition of TP restriction, there is a negative correlation between the TN:TP value and the growth rate of birch seedlings, whereas under the condition of N restriction, the TN:TP value is positively correlated with the growth rate (Ågren, 2004). This study showed that wheat yield (representing growth rate) was positively correlated with the TN:TP and TN:TK values of roots, stems plus leaves, and grain, thus further demonstrating that the farmland system was restricted by TN to varying degrees. The TC:TN and TC:TP values of leaves are usually used to indicate plant growth rate and NUE. In this study, the relationship between NUE and plant nutrients was opposite from that between yield and plant nutrients. TC:TN in stems plus leaves was positively correlated with NUE and negatively correlated with yield, similarly to the results of Xiong et al. (2020) and Chen et al. (2019). The lower the TC:TN value, the higher the plant growth rate, the higher the wheat yield, and the lower the NUE.

## Conclusions

This study demonstrated that optimized N application (N180) enhanced wheat productivity by improving nutrient allocation and the stoichiometric balance in the root-shoot-soil system. N180 treatment significantly increased the plants’ TN content and simultaneously increased the TN:TP and TN:TK ratios, thereby alleviating the N limitation in this farmland system. Moreover, yield was driven primarily by plant TN content and stem and leaf TC:TN ratios, whereas NUE was more dependent on stem and leaf TK content along with root TC:TN. N180 treatment enabled plants to better absorb and utilize nutrients, thus synergistically improving both yield and NUE. These results provide important insights for N management in wheat cultivation, particularly in N-limited farmland ecosystems. Future investigations should focus on long-term field validation of these stoichiometric relationships; examine interactions between yield and nutrient elements; and develop precision fertilization strategies based on the nutritional requirements of various plant organs.

##  Supplemental Information

10.7717/peerj.20101/supp-1 Supplemental Information 1 Original data

10.7717/peerj.20101/supp-2 Supplemental Information 2 Mountain map code

10.7717/peerj.20101/supp-3 Supplemental Information 3 Random forest code

## Additional Information and Declarations

### Data Availability

The following information was supplied regarding data availability: The original measurements and code are available in the Supplemental Files.
